# The impact of muscarinic and mGlu receptors modulators on MK-801-induced impairments in NO-dependent processes both in vitro and in vivo

**DOI:** 10.1007/s43440-025-00752-3

**Published:** 2025-06-24

**Authors:** Grzegorz Burnat, Michał Santocki, Leszek Kalinowski, Joanna M. Wierońska

**Affiliations:** 1 Maj Institute of Pharmacology Polish Academy of Sciences, 12 Smętna Street, Kraków, 31-343 Poland; 2 Department of Medical Laboratory Diagnostics— Fahrenheit Biobank BBMRI.pl, Medical University of Gdańsk, 7 Dębinki Street, Gdańsk, 80-211 Poland; 3 BioTechMed Center, Department of Mechanics of Materials and Structures, Gdańsk University of Technology, 11/12 Narutowicza Steet, Gdańsk, 80-223 Poland

**Keywords:** mGlu receptors, Muscarinic receptors, Schizophrenia, Reactive oxygen species, Superoxide dismutase, cGMP

## Abstract

**Background:**

Schizophrenia is a mental disorder with multifactorial etiology including positive, negative and cognitive symptoms. Nitric oxide (NO֗)-related biochemical pathways significantly contribute to the disease’s pathophysiology and subsequent antipsychotic treatment. Recently, metabotropic glutamatergic (mGlu) or muscarinic (M) receptors have been considered as potent antipsychotics with the potential to reverse cognitive symptoms. The aim of this study was to investigate how selected mGlu or muscarinic receptor ligands regulate the most important aspects of NO֗-related neurotransmission.

**Methods:**

In this study, MK-801—the tool compound that induces schizophrenia-related changes—was used alone or with mGlu or muscarinic receptor ligands. Positive allosteric modulators (PAM) of mGlu_2_ (LY487379), mGlu_5_ (CDPPB), M_1_ (VU0357017) and M_4_ (VU0152100) receptors were administered. cGMP levels, superoxide dismutase (SOD) activity, nitrite and GLT-1 s-nitrosilation processes were investigated in mouse brain and plasma samples, while oxidative stress was measured in vitro with the use of mouse or human astrocytic cell lines.

**Results:**

MK-801 did not change cGMP levels, while a decrease was observed in mice treated with VU0357017 or LY487379 in parallel. Increased SOD activity was observed in the cortex of MK-801-treated mice, and the compounds, with the exception of CDPPB, prevented this effect. The investigated compounds also prevented an MK-801-induced increase in plasma nitrite levels. GLT-1 protein was decreased after MK-801 treatment which was not evident in mice administered with muscarinic or mGlu ligands. GLT-1 S-nitrosilation was increased in all groups. In vitro studies revealed the potency of these compounds in counteracting MK-801-induced oxidative stress.

**Conclusions:**

The present data confirm that both mGlu and muscarinic receptor ligands may exert antipsychotic effects through biochemical pathways regulated by NO֗, in particular by decreasing oxidative stress indicators.

**Supplementary Information:**

The online version contains supplementary material available at 10.1007/s43440-025-00752-3.

## Introduction

Cognitive decline and progressive dementias are common features of many brain disorders, significantly impacting quality of life. Symptoms are diagnosed in many disease entities, very often in schizophrenia [1–4]. The main predisposing risk factors are genetic background, endothelial dysfunction, and abnormal protein aggregation [4]. The complexity and diversity of individual cases constitute a fundamental difficulty in antipsychotic treatment, which, in most cases, does not improve cognitive dysfunction.

Nitric oxide (NO﮲), a gas molecule, plays a crucial role in the pathophysiology of schizophrenia. The basic process by which NO֗ exerts its function is cyclic guanosine monophosphate (cGMP) production, which in turn regulates various physiological processes [5–10]. The first reports in this regard date back to the late 1970s, when Japanese scientist Takeo Deguchi demonstrated the relationship between cGMP cyclase activation and the presence of NO﮲ [11]. Subsequent studies confirmed the link between NO﮲ and glutamate-linked cGMP production [12, 13], as well as the correlation between NMDA receptor activation, cGMP concentration and the activity of nitric oxide synthase (NOS) [14]. Briefly, under normal conditions, NO﮲ is produced following the stimulation of NMDA receptors, which allows calcium (Ca^++^) to enter the cell and activate NOS. Besides regulating long-term potentiation (LTP) and the functioning of various proteins within neural cells [9, 15] the diverse effects of NO﮲ are critical for neurovascular homeostasis, and any dysregulation of NO﮲ signaling pathways is associated with the pathogenesis of CNS disorders, including schizophrenia [16]. In this context, NO֗ has been shown to interact with glutamatergic, dopaminergic and serotonergic pathways [17, 18]. Compared to healthy controls, reduced nitrergic neurons, NOS activity, dysregulation in the enzymes regulating the bioavalibility of L-arginine, dimethylarginine dimethylaminohydrolase 1 (DDAH1) and arginase have been observed in schizophrenia patients [19–22]. Furthermore, endothelial dysfunction, elevated oxidative or nitrosative stress, and a comprised anti-oxidant response in plasma and tissue samples from schizophrenic patients have been repeatedly demonstrated [23–25]. These may result in neurovascular unit dysfunction and blood–brain barrier hyperpermeability in schizophrenia [26], with NO֗ playing an essential role in these processes.

Available data concerning the impact of the antipsychotics currently used on NO﮲ pathways indicate that the compounds may have a detrimental effect on endothelial function and NO֗ neurotransmission [27–29]. Thus, novel antipsychotics without these disadvantages are highly needed. Animal models are key in evaluating the potency of compounds and their long-lasting neural consequences, and glutamatergic models in which NMDA antagonists produce behavioral deficits (resembling symptoms related to schizophrenia) are widely used in rodents [30–32]. MK-801, a model based on NMDA antagonist applications, has been successfully used in our laboratory [33–39]. According to the glutamatergic theory of schizophrenia, the behavioral dysfunction induced by the NMDA receptor antagonist is a consequence of the brain being flooded with glutamate; this results from decreased GABAergic inhibitory control due to NMDA receptor blockade [40, 41]. Notably, NMDA antagonists produce schizophrenia symptoms in healthy humans.

The most promising novel strategies to reverse MK-801-induced dysfunction involve stimulating metabotropic glutamate (mGlu) or muscarinic (M) receptors [42–46]. Among all the tested compounds, positive allosteric modulators of mGlu_2_, mGlu_5_, M_1_ or M_4_ receptors were mostly effective [33–39]. Recently, the effects of these compounds on NO﮲-related pathways have been investigated, and it was noticed that the mGlu receptors PAMs exaggerated MK-801’s detrimental effects on endothelial nitric oxide (eNOS) expression, but prevented methylarginine production [47, 48]. The present study constitutes the continuation of this research and focuses on other NO֗-related aspects, such as cGMP expression, oxidative stress, and S-nitrosylation processes.

## Materials and methods

### Animals

Male Albino Swiss mice (Charles River Laboratory, Sulzfeld, Germany) weighing between 20 and 25 g were used throughout this study. The animals were kept in standard laboratory conditions at room temperature (22 ± 1 °C) under a 12/12 light/dark cycle in accordance with EU Directive 2010/63/EU and subsequent regulations of the Polish Ministry of Agriculture and Rural Development (protocol no. 65/2020, 12.03.2020). Food and water were freely available. The number of animals used in each experimental group is indicated on the bar in the individual figures.

## Drugs

MK-801 and LY487379 were purchased from Tocris, Bristol, UK, while CDPPB was obtained from Abcam, Cambridge, UK. VU0357017 and VU0152100 were purchased from BioByrt, and their chemical structures and properties can be found elsewhere [49–53]. MK-801, LY487379 and VU0357017 were first dissolved in 0.9% saline, while CDPPB and VU0152100 were dissolved in 10% Tween 80. Control animals received vehicles instead of drug injections (0.9% NaCl and 10% Tween 80). The treatment groups are presented in Table 1.

For 14 days, all drugs were administered at working doses established in previous studies [33–37, 47, 48] as follows:

**Table 1:** Detailed description of the experimental groups

Groups					
Controls	MK-801 (0.3)	MK-801(0.3)/LY487379 (1)	MK-801 (0.3)/CDPPB (5)	MK-801 (0.3)/VU0357017 (10)	MK-801 (0.3) /VU0152100 (5)
	NMDA antagonist	NMDA antagonist/mGlu2 PAM	NMDA antagonist/mGlu5 PAM	NMDA antagonist/M1 AA	NMDA antagonist/M4 PAM

The compounds were administered intraperitoneally (ip) at a volume of 10 ml/kg once a day. Animals were first injected with investigated compounds and then MK-801 was administered after 30 min.

The frontal cortex (FC) and hippocampus from each animal were dissected 24 h after the last administration.

### cGMP ELISA

The samples were homogenized in 5% TCA in water, followed by centrifugation at 3000× g for 5 min. Acetylated cGMP levels in supernatant from FCs and hippocampi were quantified using an ELISA assay (Cayman Chemicals, Tallinn, Estonia, Cat# 581021) according to the manufacturer’s instructions. The standard curve range was 0.023–3 pmol/1000 µL under experimental conditions.

### Superoxide dismutase (SOD) activity assay in brain homogenates

Brain tissue samples from the FCs and hippocampi were thawed and suspended in 700 µL of PBS. Tissue homogenization was performed using a TissueLyser (Qiagen). A metal bead (Qiagen) was placed into eppendorf-type tubes, and homogenization was carried out at 23 Hz for 2 min. After homogenization, the samples were centrifuged at 1500 g for 10 min at 4 °C. The supernatant was transferred to new tubes and centrifuged again at 10,000 g for 15 min at 4 °C. The resulting supernatant was diluted and used to determine SOD. FC samples were diluted 4x, and those from the hippocampus were diluted 8x. The subsequent steps followed the manufacturer’s protocol (Thermo Fisher Sci; SOD Colorimetric Activity Kit.# EIASODC).

Additionally, protein content was quantified using the BCA method (Pierce BCA Protein Assay Kit). SOD activity was normalized to the protein concentration and expressed as percentage of control.

### Griess method

Blood was collected, transferred to sodium citrate-washed eppendorf tubes and immediately centrifuged at 1200 x g at 4 °C for 10 min. The plasma was collected into new eppendorf tubes and immediately transferred to a -80 °C freezer, where it was stored until the NO﮲ content was measured.

To enhance metabolite yield, an additional plasma deproteinization step was incorporated, following the method described by Chan et al. [54]. In this approach, the plasma sample is deproteinized using an organic solvent, which precipitates the proteins while allowing the metabolites to remain in the supernatant. For deproteinization, a 20% methanol-in-ethanol solution was prepared and mixed with the plasma sample at a ratio of 1:3. The mixture was vortexed for 1 min and incubated on ice for 20. After this, the sample was centrifuged at 20 000 x g at 4 °C for 10 min, and the supernatant containing metabolites was used to determine the concentration of NO﮲ using the Griess method.

Although NO﮲ can be measured via various direct and indirect methods, its short half-life and low in vivo concentrations make many of these methods impractical for evaluating biological samples. These challenges can be overcome by measuring stable metabolites, particularly nitrite (NO2) and nitrate (NO3). Since it is the only stable end-product of the autoxidation of NO﮲ in aqueous solutions, measuring NO2 levels provides a reliable and quantitative estimate of NO﮲ production in the in vivo environment. For this reason, an additional step was included of reducing nitrate (NO3) to nitrite (NO2) using vanadium (III) salt, as described by Brovkovych et al. [55]. This method allows for reducing NO3 and measuring NO2 in a single step, without the need to remove the reducing agent before measurement. Finally, NO2 content was quantified using a colorimetric Griess reaction.

NO3 reduction and NO2 content analysis were performed at room temperature. Briefly, a 96-well flat-bottomed plate was loaded with 50 µL of deproteinized plasma samples. Next, 50 µL of a 50 mM solution of vanadium (III) chloride (VCl3) was added to each well and mixed thoroughly. Then, 25 µL of Gress A reagent (1% sulfanilamide in 5% phosphoric H3PO4 acid; Sigma-Aldrich, Saint Louis, MO, USA) and 25 µL of Griess B reagent (0.1% N-1-(naphthyl)ethylenediamine in H2O; Sigma-Aldrich, Saint Louis, MO, USA) were added. The absorbance was measured at 540 nm using a plate reader (Thermo Scientific Multiskan Spectrum Spectrometer). NO﮲ concentration was calculated based on the NO standard curve, prepared via the serial dilution of 2 mM sodium nitrite solution (POCh, Gliwice, Poland) in phosphate-buffered saline (PBS).

### Nitro blue tetrazolium (NBT) assay in cell cultures

The intracellular production of reactive oxygen species (ROS) was measured using the nitro blue tetrazolium (NBT) reduction assay. In this test, cells are incubated with tetrazolium salt and subsequently take up NBT into their cytoplasm, where it is reduced by superoxide radicals to form purple-blue, water-insoluble formazan crystals. The formazan then releases a solubilization agent, which is quantified by measuring the absorbance of the resulting purple-blue mixture.

C8-D1A mouse astrocytes (ATCC) and 1321N1 human astrocytes (ECACC) were seeded in 96-well plates (40,000 cells per well), in DMEM cell culture medium with high glucose (Gibco)—supplemented with 10% of FBS (BioWest)—and penicillin-streptomycin solution (200 units of penicillin, and 200 µg of streptomycin per mL; Merck). After 24 h, the cells were treated with either muscarinic receptor activators, VU0357017, VU0152100, LY487379 or CDPPB. Each compound was tested in three different concentrations: 1, 5 and 10 µM. After 30 min, the cells were further treated with MK-801 (50 µM) for 24 h. Additionally, cells were stimulated with phorbol 12-myristate 13-acetate (PMA; Merck), a potent protein kinase C activator, as a positive control. After 24 h, nitro blue tetrazolium chloride (10 mg/mL; Merck) was added to cells for 1 h. After this incubation, the supernatant was discarded, and the cells were fixed with 100% methanol for 15 min at room temperature. Once fixed, the plate was left to air dry. Next, 120 µL of 2 M KOH and 140 µL of 100% DMSO were added to each well and pipetted thoroughly; the absorbance was measured at 620 nm using a Thermo Scientific Multiskan Spectrum Spectrometer [56].

### Detection of protein S-nitrosylation using biotin switch assay

Cysteine residue protein S-nitrosylation (SNO) was assessed using the biotin switch technique, as previously described by Forrester et al. [57]. Frontal cortex tissues were homogenized in 1.8 mL of lysis buffer (25 mM HEPES, 50 mM NaCl, 0.1% NP-40, 0.5 mM PMSF, and protease inhibitors, pH 7.4) using a TissueLyser II (Qiagen, Hilden, Germany) with 5 mm stainless steel beads (25 Hz, 3 min). Following centrifugation (2,000 × g, 10 min), protein concentration was determined via the BCA assay.

For SNO detection, 1.5 mg of protein homogenate was treated with 25% SDS (200 µL) and 20% methyl methanethiosulfonate (MMTS, 20 µL) in HEN buffer (250 mM HEPES, 1 mM EDTA, 0.1 mM neocuproine, pH 7.4). After incubation (50 °C, 20 min, dark), proteins were acetone-precipitated and resuspended in HENS buffer (100 mM HEPES, 1 mM EDTA, 0.1 mM neocuproine, 1% SDS, pH 8.0). Free thiols were then labeled with biotin-HPDP (Sigma-Aldrich, Darmstadt, Germany) in the presence of 80 mM sodium ascorbate (room temperature, 60 min, dark). Following another acetone precipitation, biotinylated proteins were enriched via overnight incubation with avidin–agarose beads (4 °C). Bound proteins were eluted in HENS buffer (95 °C, 10 min), centrifuged (5,000 × g, 30 s), and mixed with non-reducing Laemmli buffer. S-nitrosylation levels were analyzed by Western blot using streptavidin-HRP (Abcam #ab7403, 1:5,000). Our method’s specificity controls are provided in the Supplementary Materials, Figure S1.

The total protein (10 µg) derived from tissue lysates or 20 µL of biotin-conjugated protein eluates were separated on 8% SDS-PAGE gels and subsequently transferred onto nitrocellulose membranes following standard Western blot protocols. Post-transfer, membranes were blocked with TBS-T buffer (Thermo Scientific, Schwerte, Germany) containing 5% bovine serum albumin (Fraction V; Sigma-Aldrich, Darmstadt, Germany) for 60 min at room temperature (RT).​ Membranes were then incubated at RT for 2 h with the following primary antibodies:​ β-actin (Sigma-Aldrich, Darmstadt, Germany; Cat# A5441; dilution 1:10,000); Alzheimer precursor protein -APP (Millipore, Darmstadt, Germany; Cat# MAB348; 1:2000); Glutamate transporter 1 GLT-1(Millipore, Darmstadt, Germany; Cat# AB1783; 1:3000); and Tau (Abcam, Cambridge, UK; Cat# ab32057; 1:2000).​ Following primary antibody incubation, membranes were treated with horseradish peroxidase (HRP)-conjugated secondary antibodies at RT for 1 h:​ anti-mouse IgG (Promega; Cat# W402B; 1:5000), anti-rabbit IgG (Promega; Cat# W401B; 1:5000), and anti-goat IgG (Millipore; Cat# AP108P; 1:10,000).​ Protein bands were visualized using the SuperSignal™ West Pico PLUS chemiluminescent substrate (Thermo Scientific, Schwerte, Germany) and captured with the GeneGnome XRQ imaging system (SYNGENE, Cambridge, UK). Densitometric analysis was performed using GnomeSys software version 1.8.2. Original, unprocessed images of Western blots and biotin switch assay results are provided in the Supplementary Materials (Figures S2–S6).

### Statistical analysis

The data were checked for the normal distribution and then a one-way ANOVA followed by post-hoc comparison was performed for each dataset to determine the significance of differences between groups. A p-value of p < 0.05 was considered statistically significant. The F-statistic was reported with its corresponding degrees of freedom, presented as F(a, b), where ‘a’ denotes that between groups and ‘b’ denotes that within groups. If the normal distribution was not met, the data were analysed with a mixed-effects model.

The MK-801 group was taken as a reference group in post hoc analysis. The data are presented as means ± SEM.

## Results

### cGMP level

**Fig. 1:**
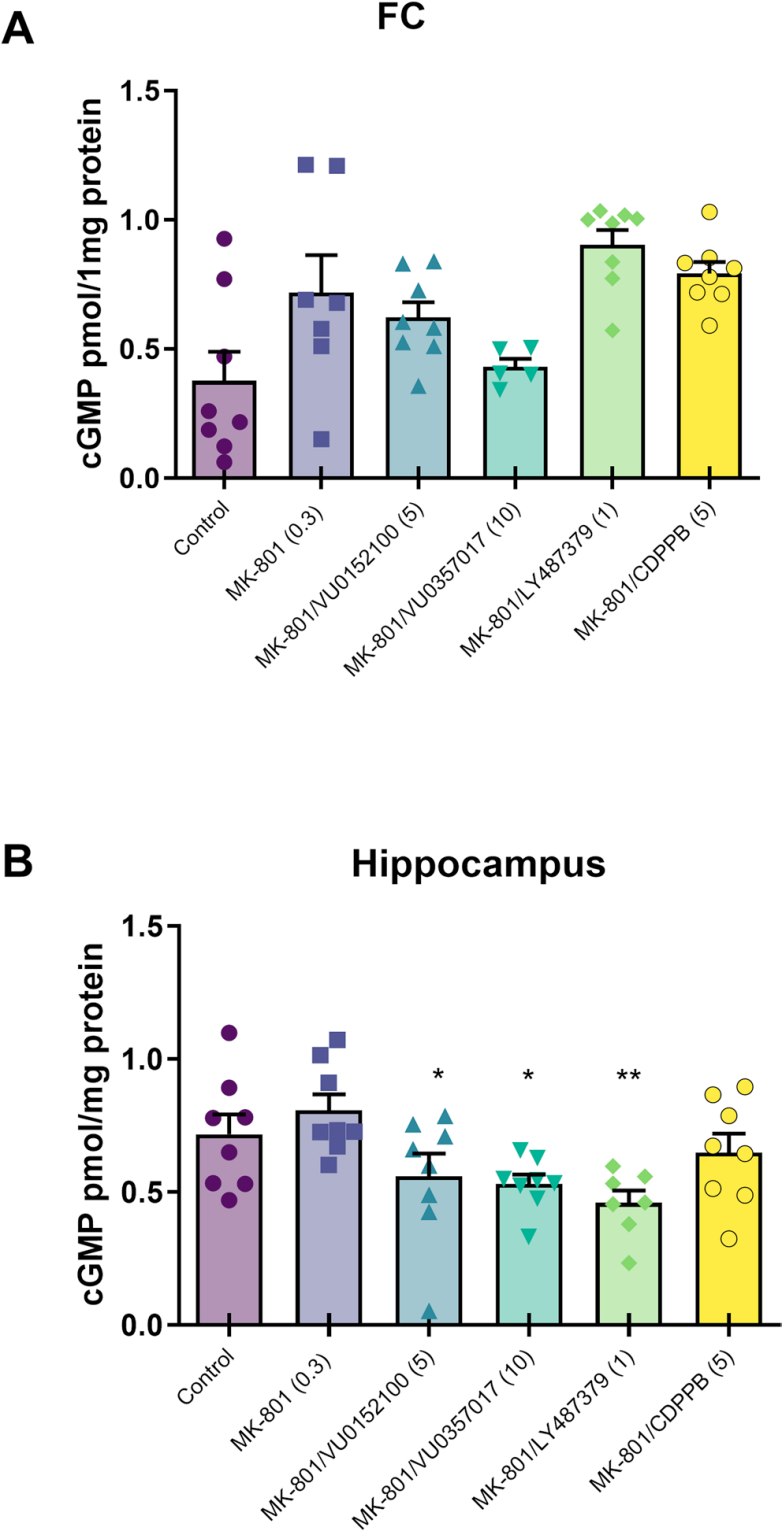
cGMP level in the frontal cortex (FC) (**A**) and hippocampus (**B**) after chronic (14-day) administration of MK-801 alone and in combination with the investigated compounds. Treatments are indicated under the bars and doses in brackets. Data are presented as means ± SEM. Dots indicate the number of animals used. * *p* < 0.01; ** *p* < 0.003 vs. MK-801

No significant changes in cGMP levels were obtained in any of the investigated brain regions following chronic MK-801 administration. Mixed-effects model analysis (REML), followed by Dunnett’s multiple comparisons, revealed that the administration of VU0152100, VU0357017, LY487379 or CDPPB did not change cGMP production in the FC compared to MK-801 (Fig. 1A). In the hippocampus, one-way ANOVA followed by Dunnett’s post hoc comparison revealed decreases in cGMP formation after the administration of VU0152100, VU0357017, and LY487379 (F_5, 41_=3.82, *p* = 0.0062, Fig. 1B).

### SOD activity in brain homogenates

**Fig. 2:**
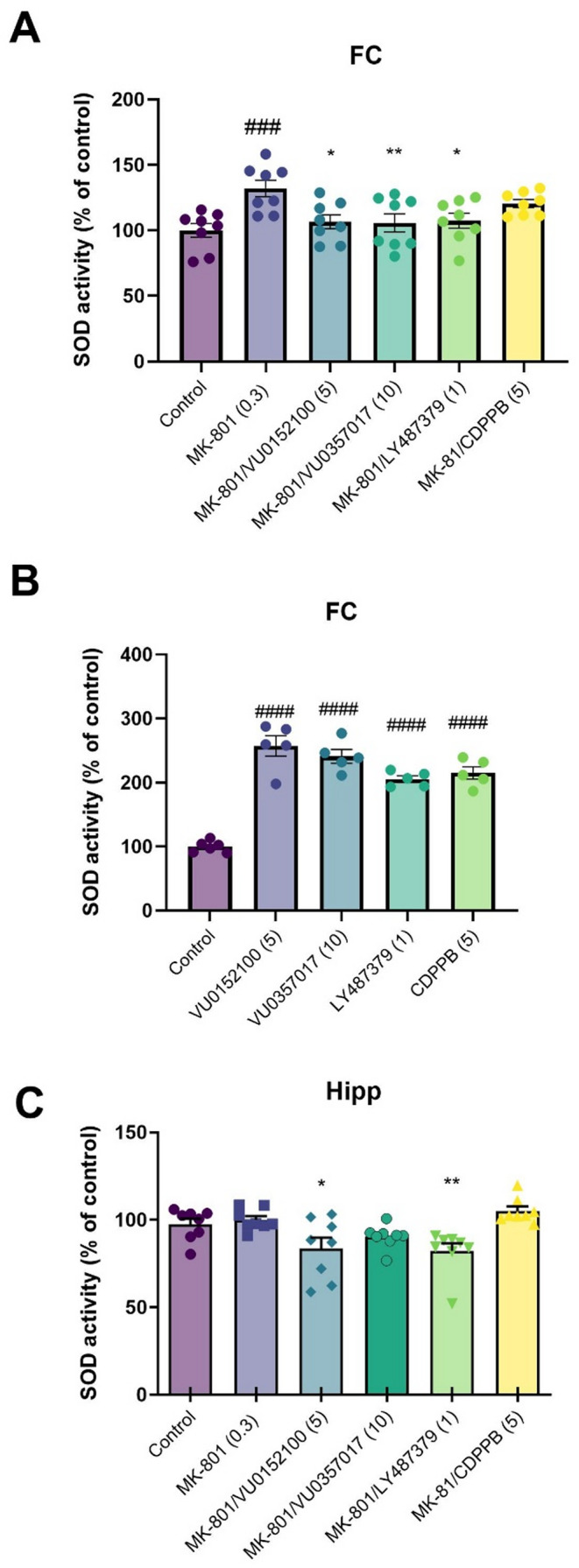
Superoxide dismutase (SOD) activity in frontal cortex (FC) (**A**, **B**) and hippocampus (**C**) after the administration of the compounds alone (**B**) or with MK-801 (**A**, **C**). Treatments are indicated under the bars and doses in brackets. Data are presented as means ± SEM. Dots indicate number of animals used. ###*p* < 0.0009 and #### *p* < 0.0001 vs. control. * *p* < 0.01; ** *p* < 0.007 vs. MK-801

A one-way ANOVA followed by Dunnett’s post hoc test revealed that MK-801 administration significantly increased SOD activity in the FC (F_5, 42_ = 4.608; *p* = 0.0019), while co-administration with VU0152100, VU0357017, or LY487379 effectively prevented this increase. In contrast, CDPPB did not exhibit any significant effect on SOD activity in the FC (Fig. 2A). When given alone, the compounds induced a significant increase in SOD activity compared to controls (F_4, 21_=44.87, *p* < 0.0001; Fig. 2B).

In the hippocampus, MK-801 administration did not result in any significant changes in SOD activity. However, treatment with VU0152100, VU0357017, or LY487379 led to a significant decrease in SOD activity compared to both the control and MK-801-treated groups (F_5, 42_ = 6.12; *p* < 0.0002), indicating a region-specific response to these compounds (Fig. 2C).

### Nitrate reduction and nitrite content analysis

**Fig. 3:**
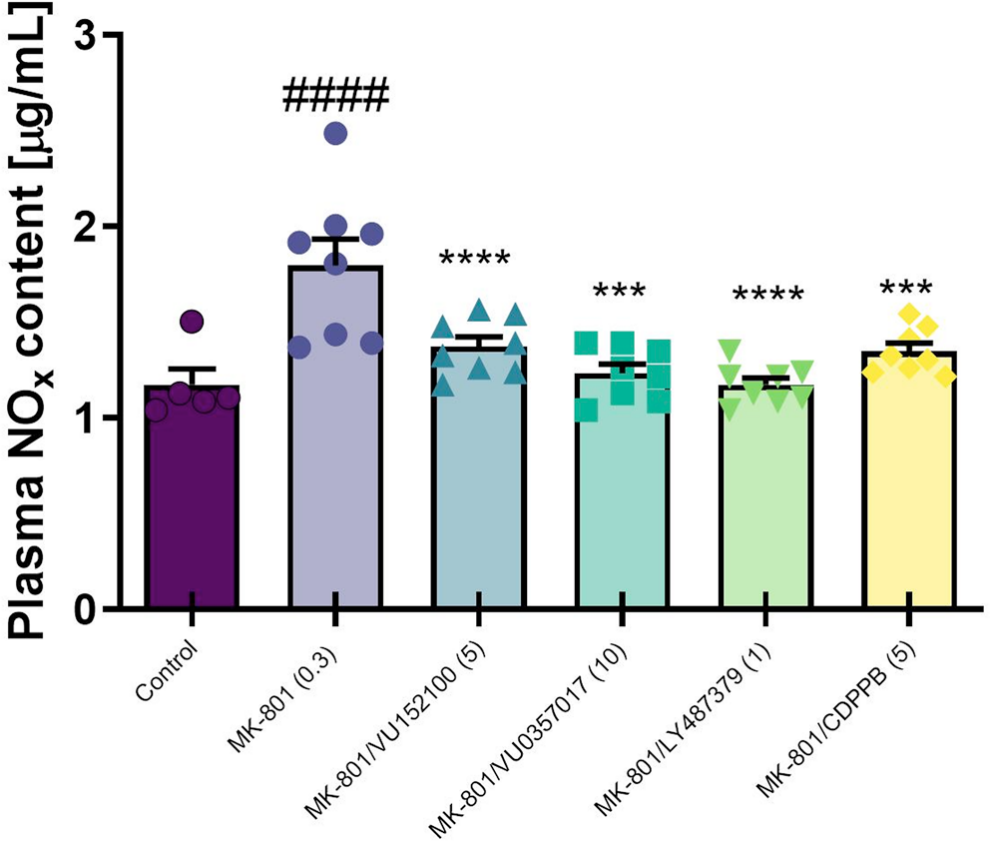
Nitrite (NOx) levels in plasma after MK-801 administration with investigated compounds. Treatments are indicated under the bars and doses in brackets. Data are presented as means ± SEM. Dots indicate number of animals used. At least ####*p* < 0.0001 vs. control and ****p < 0.0008 vs. MK-801

In plasma, MK-801 administration induced an increase in NOx levels, as calculated by one-way ANOVA. All investigated compounds significantly reversed this MK-801-induced effect (F_5, 39_=10.08, *p* < 0.0001; Fig. 3).

### NBT assay

In the C8D1A mouse astrocytic cell line, MK-801 (50 µM) induced an oxidative stress comparable to that of the reference compound PMA at 200 µM. The administration of the investigated compounds did not prevent this effect (F_6, 74_=3.789, *p* < 0.0024), as determined via one-way ANOVA.

**Fig. 4:**
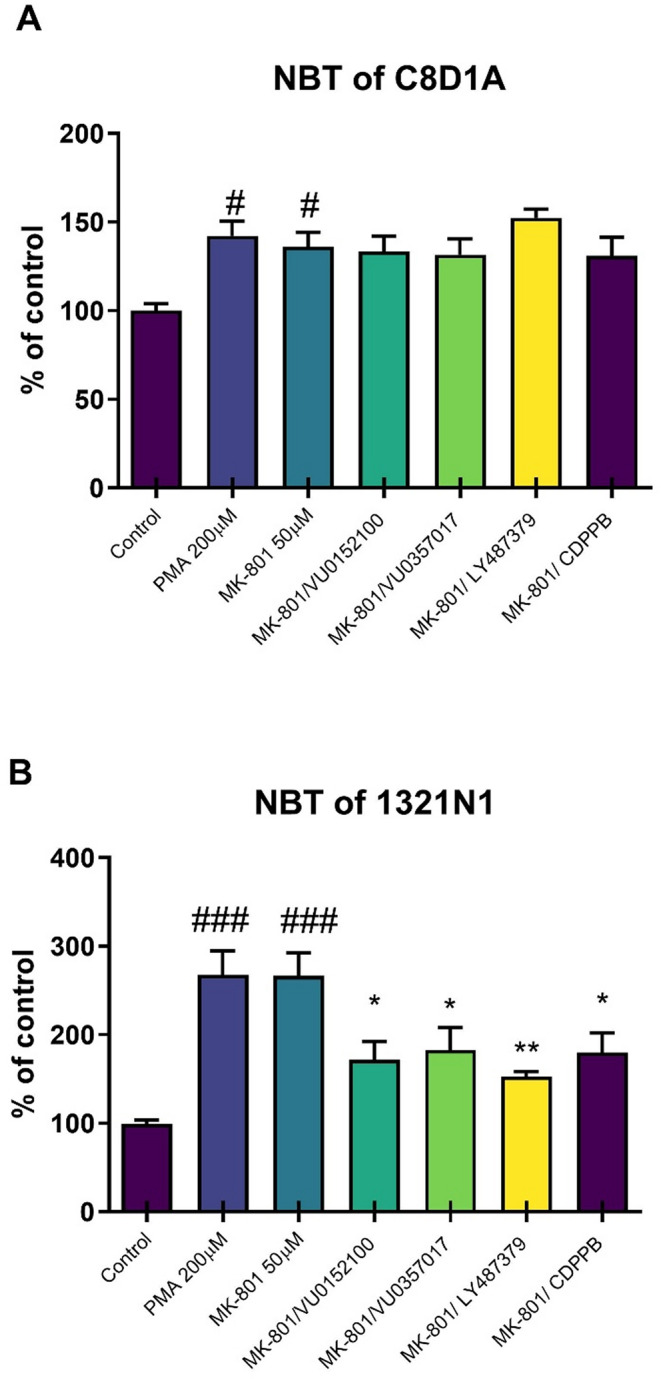
Nitro blue tetrazolium (NBT) activity in mouse (**A**) or human (**B**) astrocyte cell lines after MK-801 administration with investigated compounds (all at a concentration of 10 µM). Treatments are indicated under the bars and doses in brackets. Data are presented as means ± SEM. At least #*p* < 0.0005 and ### *p* < 0.0001 vs. control and *p < 0.03, **p < 0.003 vs. MK-801

In the 1321N1 human astrocytic cell line, MK-8001 (50 µM) also induced an oxidtive stress comparable to PMA (200 µM). One-way ANOVA revealed that all investigated compounds at all investigated concentrations prevented oxidative stress to the same extend. Here we show the results for the compounds applied at a concentration of 10 µM (F_6, 61_=8.09, *p* < 0.0001; Fig. 4).

### S-nitrosylation in brain tissue samples

Before estimating the S-nitrosylation of individual proteins, each sample was separated in polyacrylamide gel and total post-treatment S-nitrosylation was compared to that in the control group (all Western blots are presented in the Supplementary Materials). No significant differences were observed between groups.

**Fig. 5:**
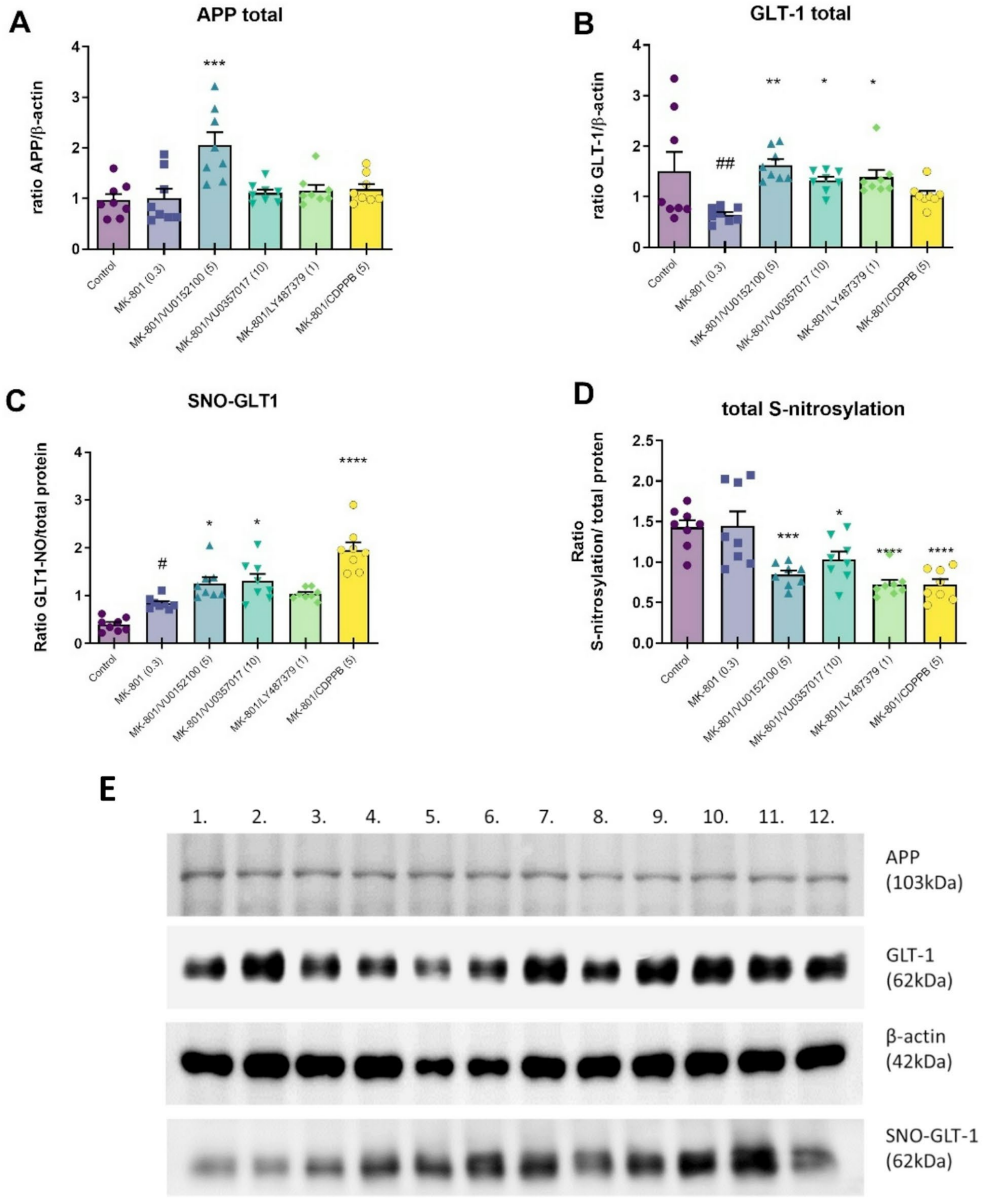
Densitometric analysis of Western blot results demonstrating changes in the expression of (**A**) Alzheimer precursor protein (APP), (**B**) glutamate transporter 1 (GLT-1), (**C**) GLT1-SNO and (**D**) total S-nitrosylation in the frontal cortex after chronic (14-day) administration of the investigated compounds. Treatments are indicated under the bars and doses in brackets. Data are presented as means ± SEM. Dots indicate number of animals used. At least #*p* < 0.02 and ##p,0.007 vs. control *p < 0.05, **p < 0.02, ***p < 0.0004 and ****p < 0.0001 vs. MK-801 group. (**E**) Representative blots

The total expression patterns of six treatment groups relative to β-actin were evaluated. No changes in the expression of Alzheimer’s precursor protein (APP) were observed following MK-801 treatment. VU0152100 administration induced an increase in APP expression, as determined with one-way ANOVA followed by Donnatt**’**s post hoc comparison (F_5, 42_ = 7.22, *p* < 0.0001, Fig. 5A), while the other compounds had no effect. This change was primarily caused by a marked reduction in β-actin levels compared to the other experimental groups. In contrast, the APP protein level remains relatively consistent across all groups.

Chronic MK-801 administration resulted in a statistically significant reduction in the expression of glutamate transporter 1 (GLT-1) relative to the control, and the administration of VU0152100, VU0357017 and LY487379 prevented this effect (F_5, 42_ = 3.93, *p* = 0.0052, Fig. 5B).

The total expression of GLT1-SNO was increased fallowing MK-801 administration, and the administration of VU0152100, VU0357017, and CDPPB exacerbated this effect (F_5, 42_=23.43, *p* < 0.0001, Fig. 5C).

Total S-nitrosylation forms were not altered by MK-801 administration, but the administration of all investigated compounds significantly decreased total S-nitrosylation (F_5, 42_=11.51, *p* < 0.0001, Fig. 5D).

## Discussion

The present study investigates the role of NO֗-related biochemical pathways in reversing schizophrenia-related deficits by mGlu or muscarinic receptor ligands. cGMP levels, oxidative stress-related indicators (SOD, NOx) and S-nitrosylation processes were examined in mouse brain samples (hippocampi or frontal cortices). The impact of the compounds on the intracellular production of reactive oxygen species (ROS) was also measured in vitro, in both human and mouse astrocytic cell lines.

NO֗-related biochemical pathways, which regulate a variety of physiological processes, are implicated in many pathological conditions, including schizophrenia and the other central nervous system (CNS) disorders [26, 58, 59].

MK-801 is widely accepted compound use to induce schizophrenia-related dysfunctions in mice [60, 61]. Hyperactivity, social withdrawal and cognitive deficits are commonly observed after MK-801 administration and the reversal of these effects may reflect antipsychotic-like activity. The stabilization of glutamatergic activity is one of the fundamental mechanisms in this process.

Recently, PAMs of muscarinic and mGlu receptors have been shown to prevent MK-801-induced behavioral changes and exert their action through different mechanisms [40, 62]. M_1_ and mGlu_5_ receptors are linked with Gq proteins and have postsynaptic localization, while M_4_ and mGlu_2_ receptors activate inhibitory Gi proteins and regulate glutamatergic release because of their presynaptic expression on glutamatergic terminals [40, 63–65]. Considering M_1_ and mGlu_5_ receptors PAMs’ mechanism of antipsychotic action, it is hypothetically very likely that a large pool of these receptors is expressed on GABAergic or, alternatively, glutamatergic neurons that innervate GABAergic neurons. Under these conditions, glutamate activity could be diminished. The inhibition of glutamate release by mGlu_2_ or M_4_ receptors PAMs is more evident, as it is directly inhibited by the activation of presynaptic receptors.

Regulation of NO֗-related pathways is critical considering the safety of the drugs and effective antipsychotic treatment. Recently, it has been shown that the tool compound MK-801 destabilized eNOS dimerization, impaired DDAH1 or PMRT5 expression, and limited L-arginine bioavailability, promoting the synthesis of its derivatives [67–69]. mGlu and muscarinic receptor PAMs improve the bioavailability of L-arginine, preventing the production of its derivatives in a MK-801 model [36, 47], while only muscarinic receptor ligands prevent MK-801-induced eNOS dysfunction [47, 48].

The present study builds upon this research, focusing on additional aspects of NO֗-related biochemistry, such as cGMP production, oxidative stress generation, and S-nitrosylation processes.

cGMP synthesis is regulated by the NMDA receptor and NO֗ [66], and the molecule serves as a key second messenger activating cGMP-dependent protein kinase and, consequently, the phosphorylation of various proteins [67]. cGMP is an important regulator of various cellular signaling pathways, playing a crucial role in learning and memory processes as part of the glutamate-NO-cGMP axis. This pathway is essential in LTP, which stabilizes learning and memory abilities [15, 68–70]. Additionally, glutamate neurotransmission/NO/cGMP pathway dysfunction has been described in schizophrenic patients [71], confirming the role of molecules in the disease.

Thus, NO/cGMP signaling cascades may be involved in the mechanism by which the NMDA antagonist induces schizophrenic symptoms. In the present study, MK-801 administration for 14 days did not alter cGMP levels in either the cortex or hippocampus, consistent with our earlier findings [38]. Other studies have reported either no changes or enhanced cGMP production following MK-801 treatment [72], but decreases in spatial learning model were also noted [34].

In the present study, changes in cGMP levels were not significant between investigated groups, while a visibly higher expression was observed in mice treated with MK-801 alone and with mGlu PAMs. In the hippocampus, decreases in cGMP production were observed in the groups treated with the investigated compounds (with the exception of the CDPPB group).

To a certain extent, these results contradict expectations. However, cGMP is produced either in endothelial cells, neurons or glia, creating technical difficulty in assessing cGMP synthesis changes in each cell type.

The cGMP neuronal pool is important in the LTP process [72], making up part of the glutamate-NO-cGMP pathway [73]. However, data in the literature concerning the impact of MK-801 on LTP demonstrate its limited impact, with no strong evidence that MK-801 destabilizes the process [74, 75]. It is likely that the inactivation of other signaling pathways, such as ERK1/2 CREB, plays a more significant role [76]. Though LTP has been most extensively studied in the hippocampus, it has been observed in a variety of other structures, including the cerebral cortex, cerebellum and amygdala [77]. The process can be NMDA-dependent or -independent [78], NOS and mGlu receptors are among the different modulators [79, 80], but the present results are far from these speculations.

However, in all possible considerations, decreased cGMP production would be a hindrance to satisfactory treatment. It is understood that the drug may have limits, and that it may be detrimental in terms of oxidative stress.

In pathological conditions involving NOS uncoupling, reactive oxygen (ROS) or nitrogen species (RNS) are generated in place of cGMP; this may underline the pathophysiology of schizophrenia [81]. Although post mortem studies are conclusive, data on oxidative stress in MK-801-induced animal models are inconsistent. It is hypothesized that the activation, rather than blockade, of NMDA receptors generates ROS, and that MK-801, as an NMDA receptor antagonist, serves as a neuroprotective agent [82]. However, some studies confirm that MK-801 may induce oxidative stress, particularly at higher doses or in an age-dependent manner (e.g., adolescent rats) [83]; this suggests that MK-801-induced ROS generation in brain tissue homogenates is ambiguous. Therefore in animal samples SOD and nitrite were investigated as the indicators of oxidative stress.

SOD accelerates the reaction of the superoxide anion (O_2_•−), a negatively charged free radical [84], forming hydrogen peroxide (H_2_O_2_) and oxygen (2O_2_•−+2 H+ → H_2_O_2_ + O_2_) [85]. Hydrogen peroxide, a product of the reaction catalyzed by SOD, can be a weak reductant or a relatively strong oxidant. While it is not a free radical, it can damage cells at low concentrations (10 µM). In the study increased SOD activity in the frontal cortex of the MK-801 group compared to controls was observed and this effect was prevented by the administration of the investigated ligands. Similar results have been obtained by other authors, who showed increased SOD activity—and other indicators of oxidative stress—in MK-801 treated rats; this was prevented by the administration of neuroprotective agents [86, 87]. In the hippocampus no changes after MK-801 administration were observed, but the investigated compounds slightly decreased its activity.

Concomitantly, SOD may serve as a double-edge sword and a key antioxidant enzyme for protecting against oxidative stress, serving as a promising therapeutic target for ROS-mediated diseases. As the investigated compounds, when given alone, increased enzyme activity, it is plausible that they act as antioxidants. We propose that MK-801-induced increase in SOD activity reflects previous cellular oxidative stress, and is a compensatory response on MK-801. Thus, in the MK-801 model, the result of their activity is to restore the redox balance.

Furthermore, MK-801-induced increases in plasma nitrite levels were also prevented by the drugs. These results suggest that both mGlu and muscarinic ligands may have the potential to prevent the MK-801-induced production of oxidative stress indicators.

To establish the ability of the investigated compounds to prevent oxidative stress, in vitro models of mouse and human astrocytic cell lines were used. MK-801-induced oxidative stress was measured as increased ROS, which was blocked by the investigated compounds in human—but not in mouse—astrocytic cells.

Finally, S-nitrosylation levels were investigated in the cortex. S-nitrosylation refers to the covalent attachment of a nitric oxide group (− NO) to a cysteine thiol within a protein, forming an S-nitrosothiol (SNO). This regulates protein function and cellular signaling by altering protein conformation, enzymatic activity, protein–protein interactions, and cellular localization. However, the exact role of S-nitrosylation is not fully understood, partly due to the transient nature of this modification. In our study, the total S-nitrosylation in brain homogenates was not altered by MK-801 administration, but the investigated compounds decreased the SNO modification of proteins. Since exaggerated SNO–protein modification may have exacerbated the etiology of schizophrenia, this property could be considered advantageous. Neither MK-801 nor the investigated compounds (except for the M1 allosteric agonist) affected the SNO-modification of APP, a key protein involved in Alzheimer’s-type dementia.

MK-801 administration decreased GLT-1 expression in the cortex, suggesting impaired glutamate uptake. This finding is consistent with the glutamatergic hypothesis of schizophrenia, which assumes that excessive glutamate activity in the cortex contributes to the disorder’s pathophysiology [40, 41].

In conclusion, the present data confirms that both mGlu and muscarinic receptor positive modulators may act through NO﮲-dependent pathways to prevent the pathological changes related to schizophrenia. However, not all effects they induce are beneficial, at least in MK-801 animal model. Although decreased cGMP production was noticed, the compounds prevented development of oxidative stress and stabilized GLT-1 protein expression. Thus, it is likely that the overall result of their action is the restoration of the redox balance and stabilization of glutamate content and reuptake.

In the context of antipsychotic activity, muscarinic ligands seem to be a slightly better choice, as mGlu activators were shown to trigger NOS uncoupling [48]. To avoid a risk of inducing adverse effects, the administration of mGlu ligands at low doses along with compounds acting at different pharmacological targets can be proposed as an optimal solution. The joint administration of mGlu and muscarinic ligands, or mGlu ligands with NO releasers, are among the good solutions [37, 47], especially that NO releasers serve as excellent antipsychotic or procognitive agents [39].

## Electronic Supplementary Material

Below is the link to the electronic supplementary material.

Supplementary Material 1

## Data Availability

No datasets were generated or analysed during the current study.

## Abbreviations

APP: Alzheimer’s precursor protein
CDPPB: 4-cyano-N-(1,3-diphenyl-1 H-pyrazol-5-yl) benzamide
cGMP: Cyclic guanosine monophosphate
CNS: Central nervous system
CREB: cAMP response element-binding protein
DDAH: dimethylarginine dimethylaminohydrolase 1
DMSO: Dimethyl sulfoxide
EDTA: Ethylenediaminetetraacetic acid
eNOS: Endothelial nitric oxide synthase
ERK1/2: Extracellular signal-regulated kinase 1/2
FC: Frontal cortex
GLT1: Glutamate transporter 1
GSNO: S-nitrosoglutathione
HEPES: 4-(2-hydroxyethyl)-1-piperazineethanesulfonic acid
HRP: Horseradish peroxidase
LTP: Long-term potentiation
LY487379: N-(4-(2-methoxyphenoxy)phenyl)-N-(2,2,2-trifluoroethylsulfonyl)pyrid-3-ylmethylamine
mGlu: Metabotropic glutamate receptors
MK-801: (5R,10 S)-(+)-5-Methyl-10,11-dihydro-5 H-dibenzo[a, d]cyclohepten-5,10-imine
MMTS: Methyl methanethiosulfonate
NADPH: Nicotinamide adenine dinucleotide phosphate
NBT: Nitro blue tetrazolium
NMDA: N-Methyl-D-Aspartate
NO: Nitric oxide
NOS: Nitric oxide synthases
O₂⁻•: Reactive superoxide
ONOO−: Peroxynitrite radical
PAM: Positive allosteric modulators
PBS: Phosphate-buffered saline
PMA: Phenyl-2-propanone
PMRT5: Protein arginine methyltransferase 5
PMSF: Phenylmethylsulfonyl fluoride
ROS: Reactive oxygen species
SDS: Sodium dodecyl sulfate
SOD: Superoxide dismutase
VU0152100: 5-{4-[2-(6-metylopirydyn-2-ylo)etylo]benzylo}-1,3-tiazolidyno-2,4-dion
VU0357017: 1-(4-methoxybenzyl)-4-oxo-1,4-dihydroquinoline-3-carboxylic acid
