# Supplementary material for: The impact of muscarinic and mGlu receptors modulators on MK-801-induced impairments in NO-dependent processes both in vitro and in vivo
Source: Pharmacol Rep. 2025 Jun 24;77(5):1309–22. doi: 10.1007/s43440-025-00752-3 (PMC12443913; doi:10.1007/s43440-025-00752-3)
Supplement: Supplementary file 1 — Supplementary Material 1 [file 43440_2025_752_MOESM1_ESM.pptx]

## Slide 1
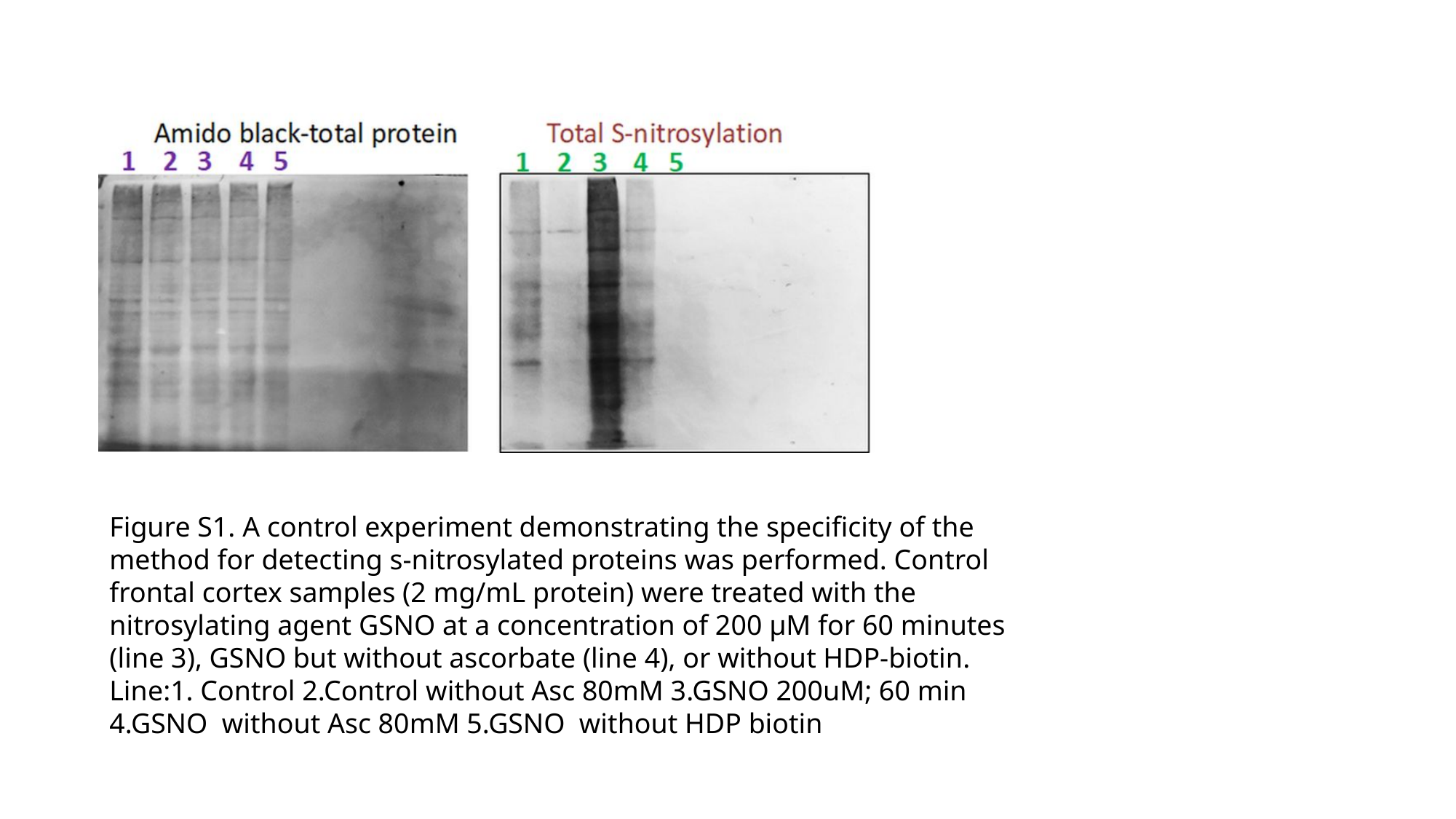

Figure S1. A control experiment demonstrating the specificity of the method for detecting s-nitrosylated proteins was performed. Control frontal cortex samples (2 mg/mL protein) were treated with the nitrosylating agent GSNO at a concentration of 200 μM for 60 minutes (line 3), GSNO but without ascorbate (line 4), or without HDP-biotin. Line:1. Control 2.Control without Asc 80mM 3.GSNO 200uM; 60 min 4.GSNO without Asc 80mM 5.GSNO without HDP biotin

## Slide 2
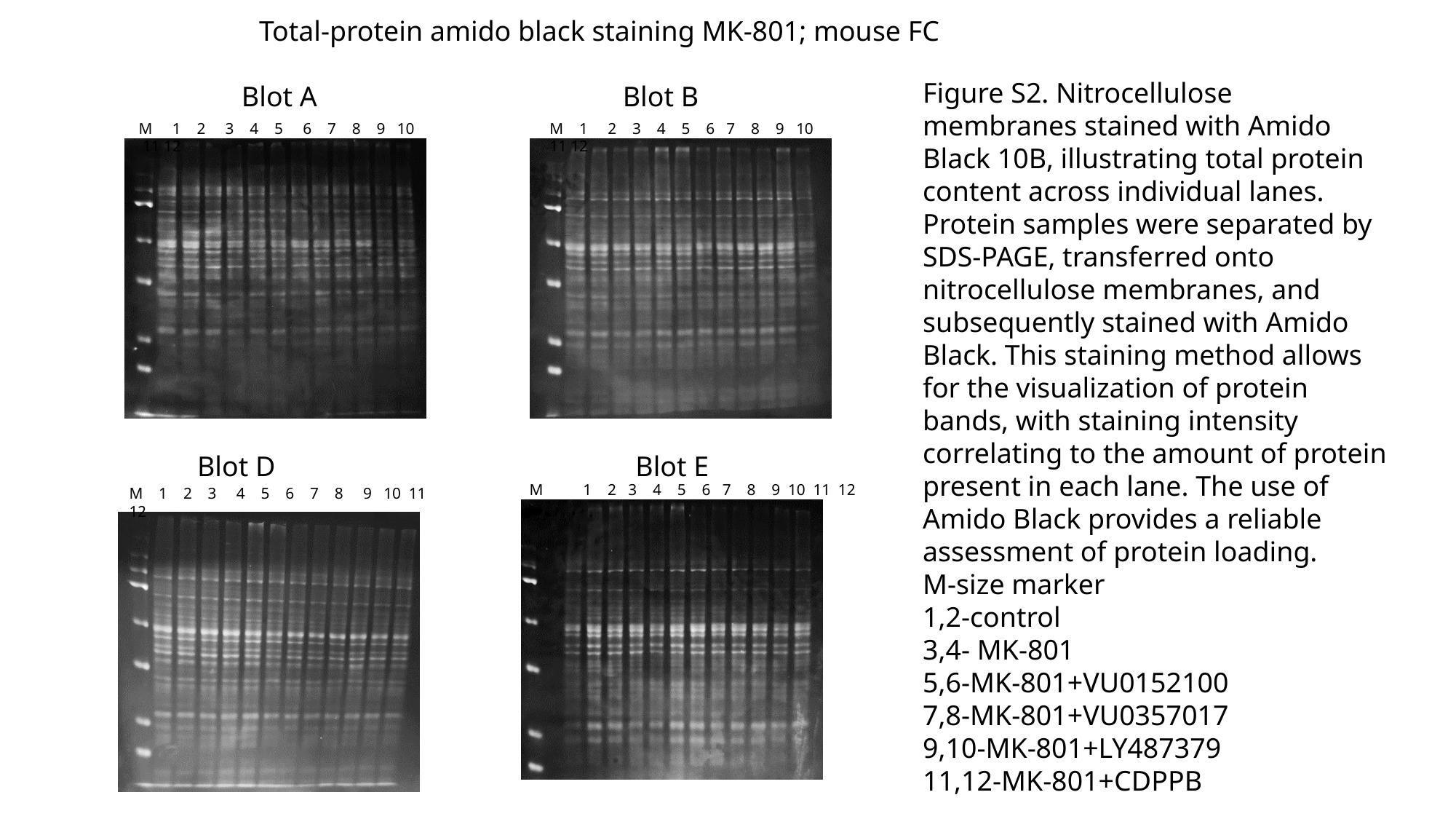

# Total-protein amido black staining MK-801; mouse FC
Figure S2. Nitrocellulose membranes stained with Amido Black 10B, illustrating total protein content across individual lanes. Protein samples were separated by SDS-PAGE, transferred onto nitrocellulose membranes, and subsequently stained with Amido Black. This staining method allows for the visualization of protein bands, with staining intensity correlating to the amount of protein present in each lane. The use of Amido Black provides a reliable assessment of protein loading.
M-size marker
1,2-control
3,4- MK-801
5,6-MK-801+VU0152100
7,8-MK-801+VU0357017
9,10-MK-801+LY487379
11,12-MK-801+CDPPB
Blot A
Blot B
M 1 2 3 4 5 6 7 8 9 10 11 12
M 1 2 3 4 5 6 7 8 9 10 11 12
Blot E
Blot D
M 1 2 3 4 5 6 7 8 9 10 11 12
M 1 2 3 4 5 6 7 8 9 10 11 12

## Slide 3
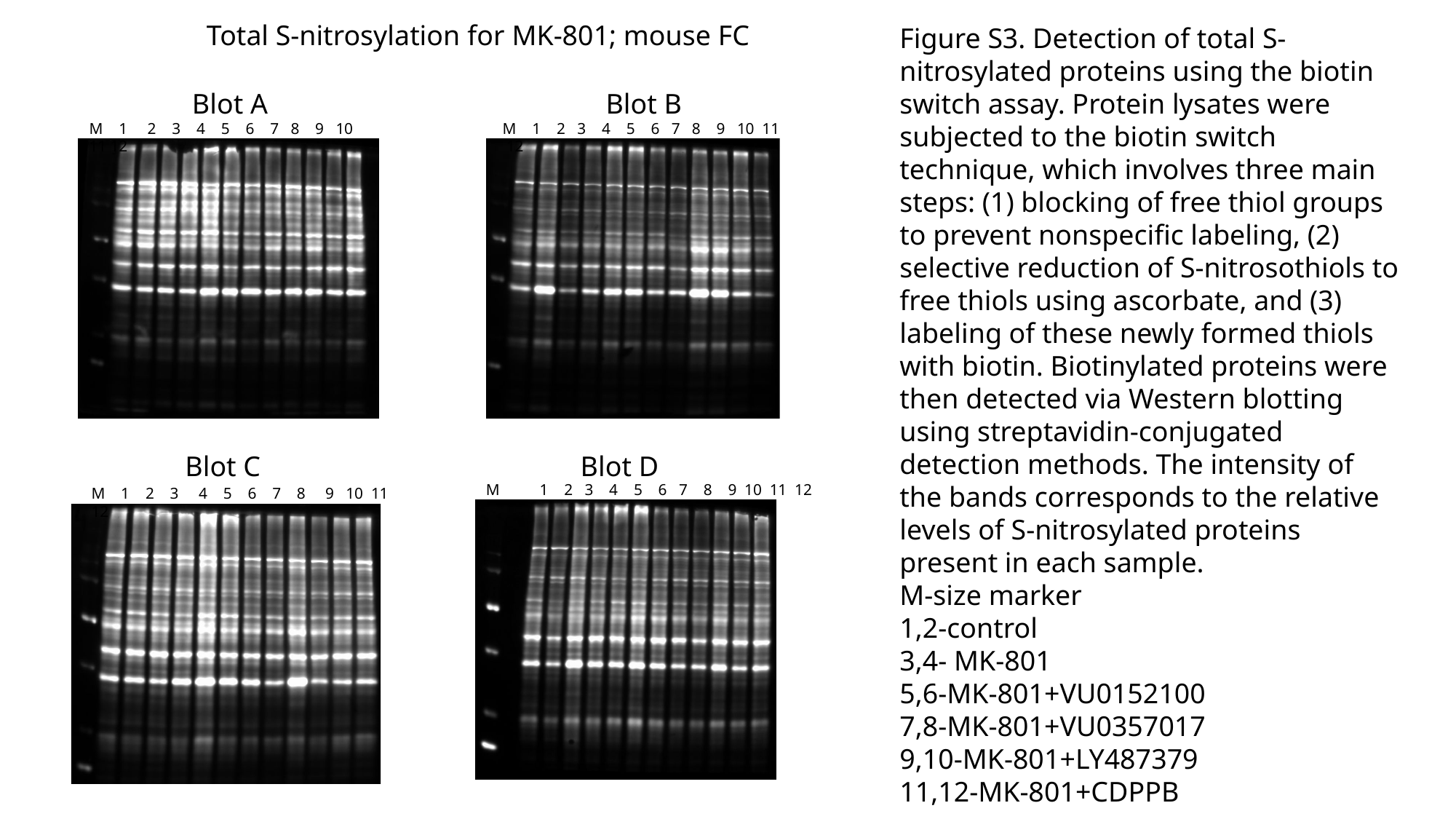

# Total S-nitrosylation for MK-801; mouse FC
Figure S3. Detection of total S-nitrosylated proteins using the biotin switch assay. Protein lysates were subjected to the biotin switch technique, which involves three main steps: (1) blocking of free thiol groups to prevent nonspecific labeling, (2) selective reduction of S-nitrosothiols to free thiols using ascorbate, and (3) labeling of these newly formed thiols with biotin. Biotinylated proteins were then detected via Western blotting using streptavidin-conjugated detection methods. The intensity of the bands corresponds to the relative levels of S-nitrosylated proteins present in each sample.​
M-size marker
1,2-control
3,4- MK-801
5,6-MK-801+VU0152100
7,8-MK-801+VU0357017
9,10-MK-801+LY487379
11,12-MK-801+CDPPB
Blot A
Blot B
M 1 2 3 4 5 6 7 8 9 10 11 12
M 1 2 3 4 5 6 7 8 9 10 11 12
Blot D
Blot C
M 1 2 3 4 5 6 7 8 9 10 11 12
M 1 2 3 4 5 6 7 8 9 10 11 12

## Slide 4
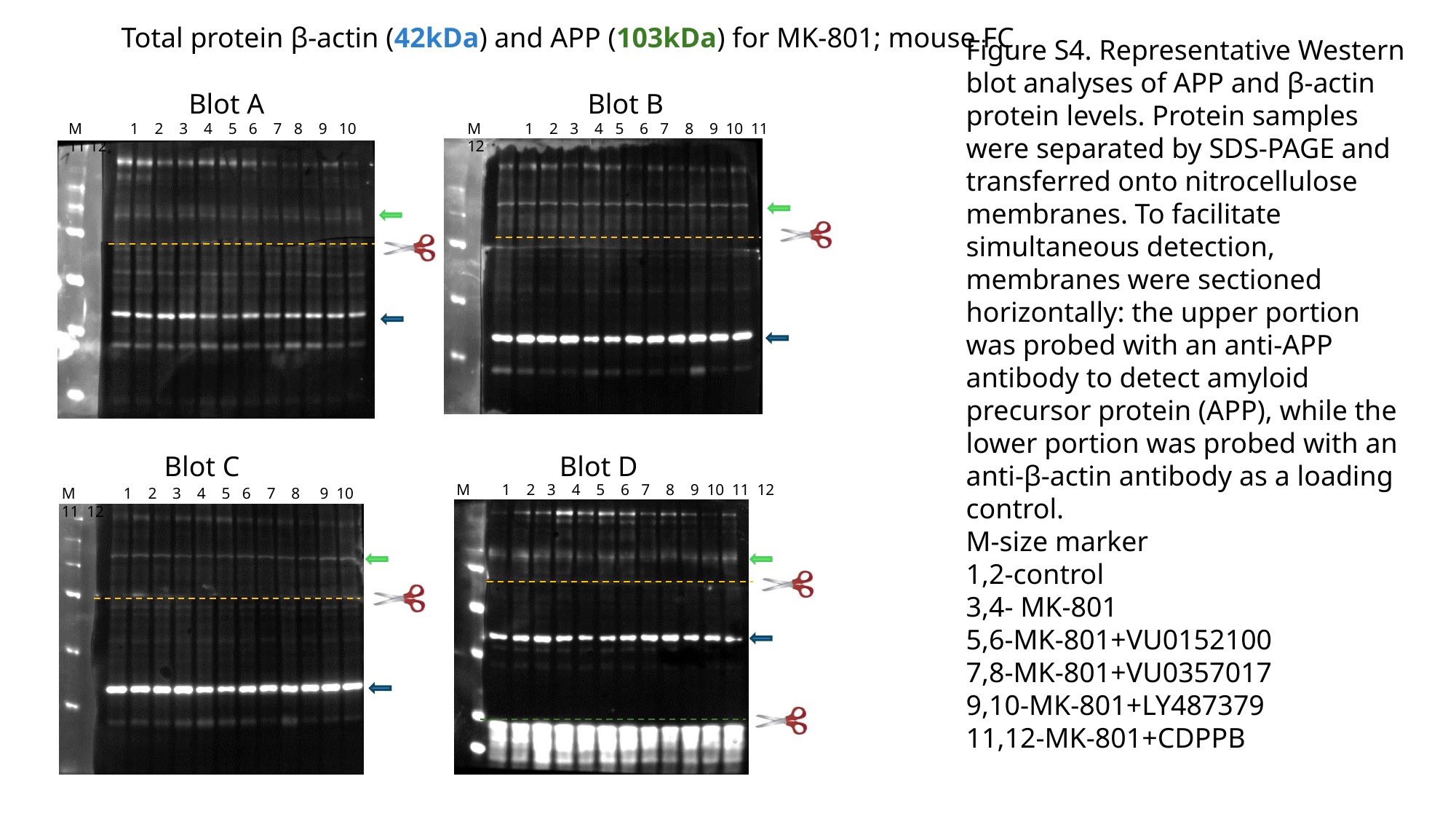

# Total protein β-actin (42kDa) and APP (103kDa) for MK-801; mouse FC
Figure S4. Representative Western blot analyses of APP and β-actin protein levels. Protein samples were separated by SDS-PAGE and transferred onto nitrocellulose membranes. To facilitate simultaneous detection, membranes were sectioned horizontally: the upper portion was probed with an anti-APP antibody to detect amyloid precursor protein (APP), while the lower portion was probed with an anti-β-actin antibody as a loading control.
​M-size marker
1,2-control
3,4- MK-801
5,6-MK-801+VU0152100
7,8-MK-801+VU0357017
9,10-MK-801+LY487379
11,12-MK-801+CDPPB
Blot A
Blot B
M 1 2 3 4 5 6 7 8 9 10 11 12
M 1 2 3 4 5 6 7 8 9 10 11 12
Blot D
Blot C
M 1 2 3 4 5 6 7 8 9 10 11 12
M 1 2 3 4 5 6 7 8 9 10 11 12

## Slide 5
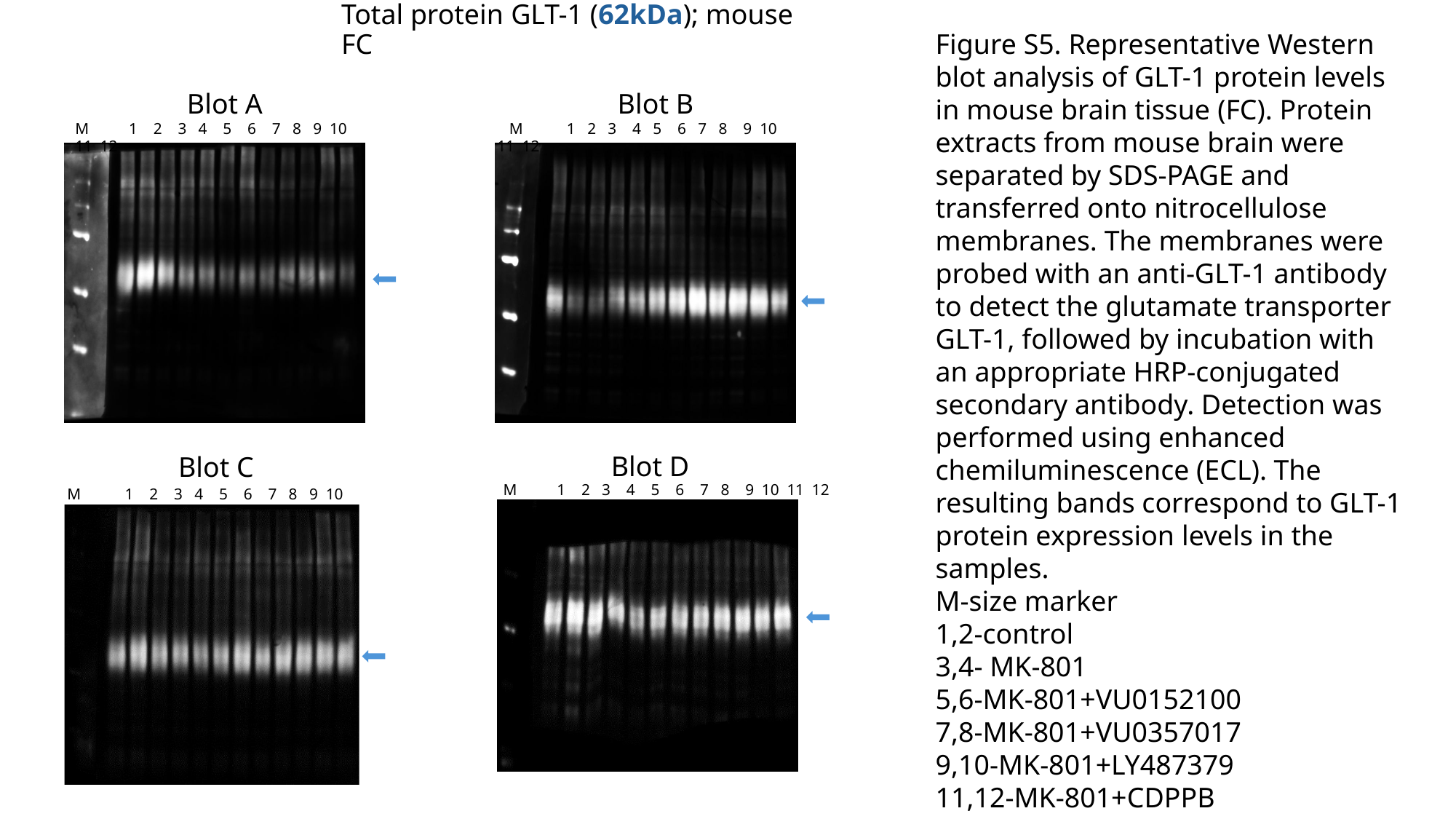

# Total protein GLT-1 (62kDa); mouse FC
Figure S5. Representative Western blot analysis of GLT-1 protein levels in mouse brain tissue (FC). Protein extracts from mouse brain were separated by SDS-PAGE and transferred onto nitrocellulose membranes. The membranes were probed with an anti-GLT-1 antibody to detect the glutamate transporter GLT-1, followed by incubation with an appropriate HRP-conjugated secondary antibody. Detection was performed using enhanced chemiluminescence (ECL). The resulting bands correspond to GLT-1 protein expression levels in the samples.
M-size marker
1,2-control
3,4- MK-801
5,6-MK-801+VU0152100
7,8-MK-801+VU0357017
9,10-MK-801+LY487379
11,12-MK-801+CDPPB
Blot A
Blot B
M 1 2 3 4 5 6 7 8 9 10 11 12
 M 1 2 3 4 5 6 7 8 9 10 11 12
Blot D
Blot C
M 1 2 3 4 5 6 7 8 9 10 11 12
M 1 2 3 4 5 6 7 8 9 10 11 12

## Slide 6
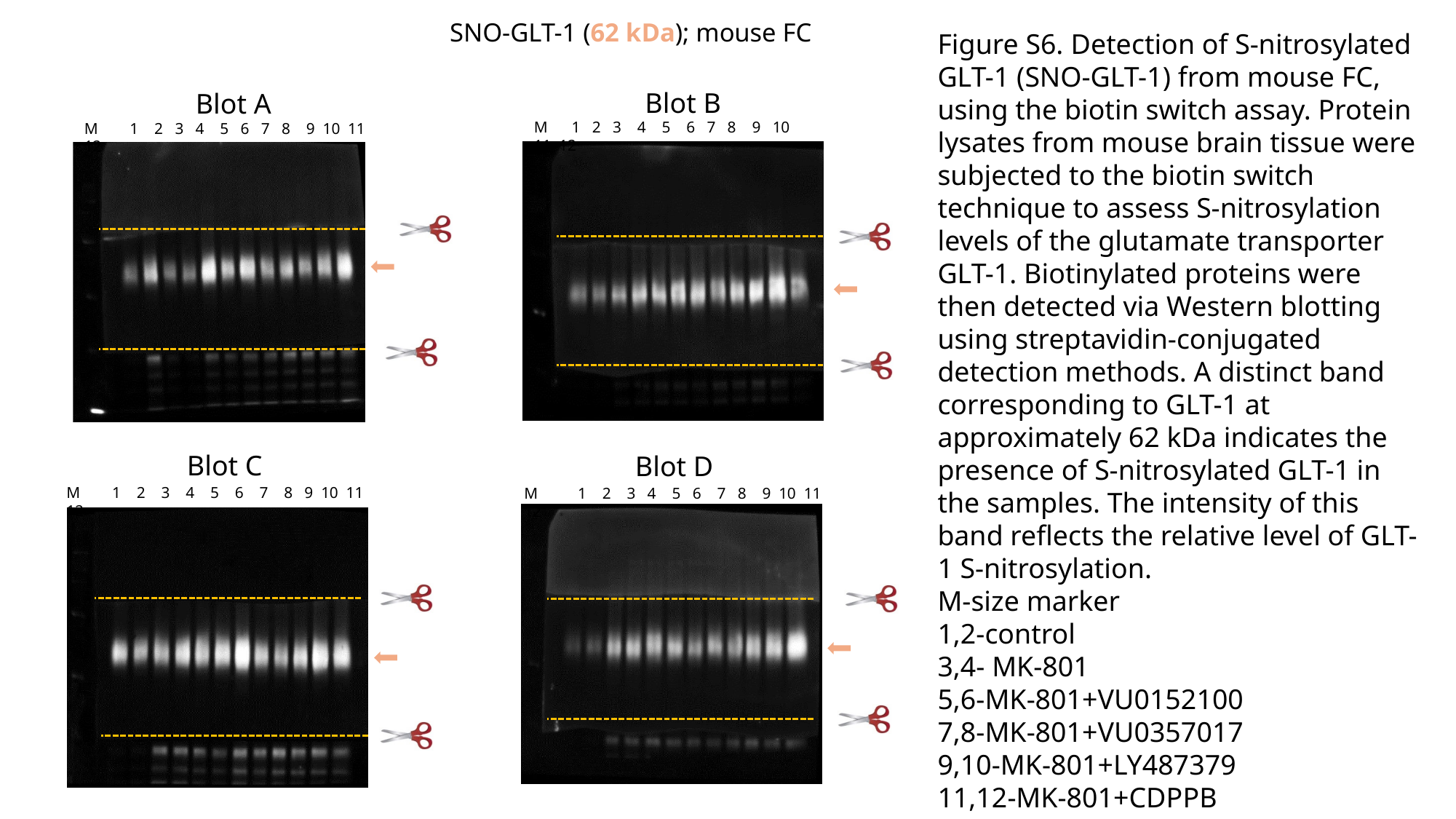

# SNO-GLT-1 (62 kDa); mouse FC
Figure S6. Detection of S-nitrosylated GLT-1 (SNO-GLT-1) from mouse FC, using the biotin switch assay. Protein lysates from mouse brain tissue were subjected to the biotin switch technique to assess S-nitrosylation levels of the glutamate transporter GLT-1. Biotinylated proteins were then detected via Western blotting using streptavidin-conjugated detection methods. A distinct band corresponding to GLT-1 at approximately 62 kDa indicates the presence of S-nitrosylated GLT-1 in the samples. The intensity of this band reflects the relative level of GLT-1 S-nitrosylation.
M-size marker
1,2-control
3,4- MK-801
5,6-MK-801+VU0152100
7,8-MK-801+VU0357017
9,10-MK-801+LY487379
11,12-MK-801+CDPPB
Blot B
Blot A
M 1 2 3 4 5 6 7 8 9 10 11 12
M 1 2 3 4 5 6 7 8 9 10 11 12
Blot C
Blot D
M 1 2 3 4 5 6 7 8 9 10 11 12
M 1 2 3 4 5 6 7 8 9 10 11 12
